# Post-hospital syndrome in adults with asthma: a case-crossover study

**DOI:** 10.1186/1710-1492-9-49

**Published:** 2013-12-23

**Authors:** Mohsen Sadatsafavi, Larry D Lynd, J Mark FitzGerald

**Affiliations:** 1 Department of Medicine, Institute for Heart and Lung Health, The University of British Columbia, 7th Floor, 828 West 10th Avenue, Research Pavilion, Vancouver V5Z 1 M9, Vancouver, BC, Canada; 2 Collaboration for Outcomes Research and Evaluation, Faculty of Pharmaceutical Sciences, the University of British Columbia, Vancouver, Canada; 3 Centre for Health Evaluation and Outcome Sciences, the University of British Columbia, Vancouver, Canada

## Abstract

**Background:**

Post-hospital syndrome refers to the period of generalized risk of adverse health outcomes among patients who are recently discharged from hospital. This period is associated with a short-term increased risk of readmission which may not be related to the original condition. While the majority of studies of post-hospital syndrome have focused on all-cause readmissions, whether and to what extent such a phenomenon exists within discrete medical conditions is not yet known.

**Objective:**

To investigate whether the risk of admission due to asthma is increased in individuals who are discharged following any-cause hospital admission.

**Methods:**

Using administrative health data for the period 1997 to 2007 from the province of British Columbia, Canada, we created a cohort of adults with asthma. Using a case-crossover design, we assessed the association between discharge from a hospital (exposure) within 30 days before an asthma-related hospitalization (the outcome), using two 30-day control periods within the same subject. Conditional logistic regression was performed to calculate the relative risk (RR) of the outcome in association with exposure. We performed several sensitivity and subgroup analyses.

**Results:**

The final cohort included 3,852 patients experiencing 6,333 instances of the outcome. Mean age at the time of the outcome was 43.7 (SD 14.2), 69.0% of such outcomes belonged to females. The RR of the outcome within the next 30 days of a previous any-cause discharge was 1.40 (95% CI 1.22 - 1.59). However, the association was mainly caused by discharge from asthma-related admission [RR = 1.99 (95% CI 1.65 - 2.39)]. The RR associated with non-asthma-related discharge was 0.88 (95% CI 0.74 - 1.04) and was not statistically significant. Similar results were obtained in a range of sensitivity analyses.

**Discussion:**

Our results indicate that in patients with asthma, the 30-day risk of asthma-related admission is increased after an episode of asthma-related hospitalization, but not after an episode of non-asthma-related hospitalization.

## Introduction

Post-hospital syndrome refers to the acquired, transient period of generalized increased risk for a broad range of health conditions after discharge from hospital [1]. An important manifestation of this syndrome is the high rate of readmission in the critical 30 days after discharge, which is not necessarily due to the same condition. A recent study showed that nearly 20% of patients covered by a US national insurance program (Medicare, consisting of individuals aged 65 and older as well as those with disabilities or end stage renal disease) discharged from a hospital had another acute medical problem within the subsequent 30 days that necessitated re-hospitalization [2]. Reasons for readmissions often include heart failure, pneumonia, COPD, infection, gastrointestinal conditions, mental illness, metabolic derangements, and trauma [2]. Various etiological reasons are postulated for this phenomenon, including disturbances of sleep, nutritional issues, pain and discomfort, and psychological confusion [1].

While the typical post-hospital syndrome affects mostly elderly patients with co-morbid conditions, the potential etiologic factors exist regardless of age. In addition, potential causal factors might have differential effects across different medical conditions. As such, studying post-hospital syndrome within the realm of specific diseases can be informative from a patho-physiological perspective, and is also important from a clinical perspective as it can help risk-stratify individuals at the time of discharge from hospital.

To our knowledge, no previous study has examined the risk of an asthma-related hospitalization after discharge from a previous hospitalization. Any hospitalization independent of the cause might affect the risk of a subsequent asthma exacerbation in complex ways. Some of the postulated factors for the post-hospital syndrome can increase the risk of worsening of asthma symptoms and asthma exacerbations. For example, psychological stress is known to be associated with asthma attacks [3], and changes in the immune system and malnutrition are all associated with an increased risk of the worsening of asthma [4]. In addition, medications that patients receive during admission (e.g., non-steroidal anti-inflammatory drugs or beta-blockers) might cause drug-induced exacerbations [5]. On the other hand, patients with asthma who are hospitalized for other reasons will most likely also be managed for their asthma during the inpatient period, and at times receive potent anti-inflammatory medications which can reduce the risk of an asthma attack. Combining all these factors, there seems to be different potential mechanisms for post-hospital syndrome affecting the risk of an asthma-related admission that has not hitherto been evaluated.

Using administrative health data from the province of British Columbia, Canada, we set out to evaluate whether individuals with asthma are at higher risk of an episode of asthma-related hospitalization after discharge from hospitalization due to any cause. We also evaluated such a risk across different subgroups of patients, and evaluated the risk according to the cause of the original admission as being asthma-related, non-asthma respiratory-related, or non-respiratory-related.

## Methods

### Data

Records of the utilization of all billed services for the fiscal years 1997 to 2007 were obtained from the British Columbia Ministry of Health (http://www.popdata.bc.ca/data) [6]. This study was approved by University of British Columbia-Providence Health Care Research Ethics Board (#H08-01287). No consent was required as the data consisted of anonymized health records released to investigators in accordance with the Provincial Freedom of Information and Protection of Privacy Act. We had access to consolidation files [7] and all records of inpatient [8] and outpatient [9] encounters, as well as medication dispensations (the PharmaNET database [10]) during this period. Hospitalization and outpatient service use records contain encounter dates and International Classification of Diseases (ICD, 9^th^ and 10^th^ revisions) codes for the reason for the encounter. For hospitalization records, up to 25 ICD codes are recorded, one of which is designated as the ‘most responsible’ diagnosis; i.e., the diagnosis responsible for the greatest portion of the patient’s stay in the hospital. The length of stay in the hospital as well as admission type (urgent versus elective) is also available. The medication dispensation database includes variables such as the unique drug identifier and date of dispensation [11].

### Study cohort

Adults (age 18 years and older) were considered as having asthma if during a rolling time window of 12 months they filled prescriptions for at least three asthma-related medications (list of such medications is available in Additional file 1). The date of the first of the three prescriptions was considered the cohort *entry date*. The date of the last resource use of any type was considered the *exit date*.

### Design

**Figure 1:**
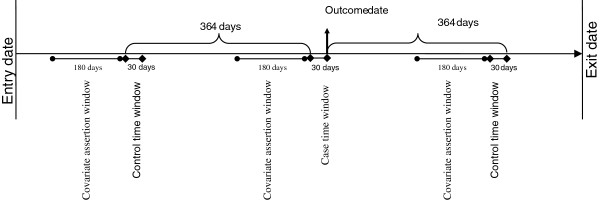
Schematic illustration of the cohort construction and analysis type. The arrow from left to right represents the timeline of an individual within the data. The vertical arrow shows an outcome date (admission to hospital due to asthma). The immediate 30-day prior to this date constitutes the case time window. For each subject, up to two control time windows of the same length were also selected, each 364 days before and after the start of the case time window. The presence of discharge from a hospitalization in the case and control-time windows defines the exposure.

We chose a case-crossover design for this study in which each individual subject acts as their own control [12]. In the study of the association of transient exposures with acute outcomes, such as this study, the case-crossover design is an attractive option as it inherently removes the potential biasing effects of unmeasured, time-invariant confounding factors [13]. Adjusting for such unmeasured confounding factors is particularly important in the present context as both the exposure and outcome in this study are hospitalization events, and many factors might affect the overall person-specific rate of hospitalization (e.g., the patient’s and care provider’s threshold for inpatient care, the availability of hospital beds in the local health area, and co-morbid conditions). This design is similar to the classic case–control design, with the main difference that the case and control periods belong to the same subject, albeit at different times. In a sensitivity analysis we also performed a conventional nested case–control study to evaluate the robustness of the results to the design specifications. A schematic illustration of the case-crossover design is provided in Figure 1.

### Exposure

The primary exposure in this analysis was discharge from an episode of hospital admission with at least one full day of stay, regardless of the cause. We further categorized such admissions to be due to asthma-related versus non-asthma-related as well as respiratory-related versus non-respiratory-related. Asthma-related admissions were those with the most responsible diagnosis being for asthma (ICD-9 493.xx, ICD-10 J45/J46). Respiratory-related admissions were those with the most responsible diagnosis being for a respiratory condition (ICD-9 codes 460–519, ICD-10 codes Jxx).

### Outcome

The outcome of interest was a non-elective admission to hospital with the main diagnosis being asthma (as described above). A national chart review of the data for the 2005–2006 fiscal year showed that the main diagnosis of asthma in a discharge record had a sensitivity of 87% (95% CI 79%–95%) and a positive predictive value of 90% (95% CI 85%–95%) [14]. In addition, restricting the study population to those who satisfied a case definition of asthma means that the subset of hospitalizations included in the analysis were even more likely to be truly asthma-related. In line with the general definition of post-hospital syndrome, readmissions that counted towards the outcome did not include elective admissions or emergency room visits that did not result in inpatient admissions.

### Case and control time windows

The 30-day period immediately before each asthma-related hospitalization was considered as the case time window (see Figure 1). For each case time window, we considered up to two control time windows ending exactly 364 days before and after the index date. The choice of the timing of control time windows was to adjust for the effect of seasonality as well as day of the week as such factors might be potentially correlated with the risk of hospitalizations. We specifically avoided using control time windows that are adjacent to case time windows because first, the individual is protected from experiencing a hospitalization event in the time period immediately after the case time window (due to the length-of-stay of the index hospitalization associated with the case time window); second, the time adjacent to the case time window would belong to different month and potentially season which is an important factor affecting the risk of asthma-related hospitalization.

Eligible control windows were those that fit entirely within the interval between the entry and exit date of the individual. Control time windows that did not satisfy such criteria were removed. Case time windows for which both control time windows were excluded were also excluded as they could not contribute to the statistical inference. Each individual could contribute several cases and associated control time windows.

### Analysis

We calculated the rates of the occurrence of exposure in both the case and control time windows. In doing so, and in line with basic principles, we weighted each control time window according to the reciprocal of the number of control time windows for the corresponding case time window [15]. In the main analysis, using conditional logistic regression we calculated the adjusted relative risk (RR) of the asthma-related hospitalization (outcome) in association with exposure. We controlled for potentially time-varying measures of asthma severity (number of asthma-related admissions, outpatient service use, medication dispensation, as well the number of dispensed canisters of short-acting beta-agonists (SABA), inhaled corticosteroids (ICS), and combined ICS and long-acting beta-agonists (ICS + LABA)) and general measures of co-morbidity (Charlson co-morbidity index [16], total number of admissions, outpatient services use and medication dispensations), all measured in the 180 days prior to the (Case and control) time window (Figure 1). Robust variance estimators were used for inference to account for within-subject clustering of events (as outcomes that belong to the same person cannot be considered as independent observations) [17].

### Subgroup, sensitivity, and alternative analyses

Subgroup analysis involved separately performing the analysis by sex and age groups. We performed several sensitivity analyses. These included an unadjusted analysis as well as performing the analysis using a conventional nested case–control design. The nested case control design followed the same principles as the main study (cohort definition, inclusion criteria, case and control time windows) with the difference being that the control time windows were selected from other individuals in the risk set, and no 364-day-interval rule was applied. For a given case time window, the risk set was defined as those individuals who were at the risk of experiencing the outcome (that is, the follow-up day of the index case they was between the entry and exit date of the controls) and had the same sex, year of birth, and similar entry date (within 180 days) as the case. The nested-case control analysis was further adjusted for all covariates that were controlled for in the main case-crossover design. Other sensitivity analyses included choosing control time windows at different distances from the case time window. We performed two alternative analyses exploring the association between discharge from hospital and 60-day and 90-day risk of asthma-related admission. Finally, to evaluate the potential impact of treatment in the post-discharge period in the association between the exposure and outcome, we performed an additional sensitivity analysis in which the regression model was further controlled for whether the individual received any controller medication (systemic corticosteroids, inhaled corticosteroids with or without long-acting beta-agonists, or leukotriene receptor antagonists) during the exposure (case or control) time window.

## Results

The study cohort constituted 178,192 individuals with asthma among whom there were 6,333 asthma-related hospitalizations (outcome) experienced by 3,852 unique individuals. The associated case time windows for these events were matched to 10,737 control time windows (1.70 control time windows per event). Table 1 provides the basic demographics characteristics of the individuals in the final analysis.

**Table 1:** Characteristics of individuals in the final data set

**Number of outcomes per person**	
**Mean (S D)**	**1.64 (1.81)**
**[1,2,3,4+]**	**[2804,575,212,261]**
Entry time	
(In years since 1/1/1997)	**1.5 (2.2)**
Event time	
(In years since 1/1/1997)	**5.8 (3.0)**
Event time	
(Since entry)	**4.3 (2.8)**
Age at entry date	
Mean (SD)	**39.4 (14.1)**
Age at outcome date	
Mean (SD)	**43.7 (14.2)**
Sex	
Female	**69.0%**
Male	**31.0%**

Results of the conditional logistic regression are provided in Table 2. In 10.8% of case time windows there was a discharge from hospital. In comparison, in 7.9% of control time windows there was a discharge from hospital, resulting in a RR of asthma-related re-hospitalization following a hospital admission from any cause of 1.40 (95% CI 1.22 - 1.59), P < 0.001. Nevertheless, such an increased risk of the outcome was mainly due to the asthma-related discharge, with an RR of 1.99 (95% CI 1.65 - 2.39). Non-asthma-related discharges were not associated with 30-day asthma-related readmissions (RR = 0.88 (95% CI 0.74 - 1.04), P = 0.14). 81% of all respiratory-related discharges were due to asthma. As such, and expectedly, they were associated with an increased risk of the outcome (RR = 1.89 (95% CI 1.60 - 2.22)). Respiratory-related, non-asthma discharges were not associated with the risk of the outcome (RR = 1.23 (95% CI 0.90 - 1.68), P = 0.20). Non-respiratory-related admissions were associated with a lower risk of the outcome (RR = 0.78 (95% CI 0.63 - 0.96)).

**Table 2:** Association between exposure (discharge from hospital) and outcome (asthma-related admission in the next 30 days)

**Exposure**	**Frequency**		**Adjusted RR**	**P-value**
	**Case window**	**(95% CI)†Control window**		
	**N = 6,333**	**N = 10,737**		
Any discharge	687 (10.8%)	849 (7.9%)	1.40 (1.22 – 1.59)	<0.001*
Asthma-related discharge	442 (7.0%)	389 (3.6%)	1.99 (1.65 – 2.39)	<0.001*
Non-asthma-related discharge	273 (4.3%)	495 (4.6%)	0.88 (0.74 – 1.04)	0.141
Respiratory-related discharge	523 (8.3%)	497 (4.6%)	1.89 (1.60 – 2.22)	<0.001*
Respiratory-related, non-asthma discharge	89 (1.4%)	124 (1.2%)	1.23 (0.90 – 1.68)	0.199
Non-respiratory-related discharge	189 (3.0%)	381 (3.5%)	0.78 (0.63 – 0.96)	0.017*

*Significant at 0.05 level.

†All RRs are adjusted for the following variables estimated in the 180 days prior to the start of the time window: number of asthma-related hospital admissions, outpatient services use, medication dispensations, number of dispensations of short-acting beta-agonists, inhaled corticosteroids, and combined inhaled corticosteroids and long-acting beta-agonists, as well as total number of hospital admissions, outpatient services use, medication dispensations, and the Charlson co-morbidity index.

### Subgroup analyses

**Figure 2:**
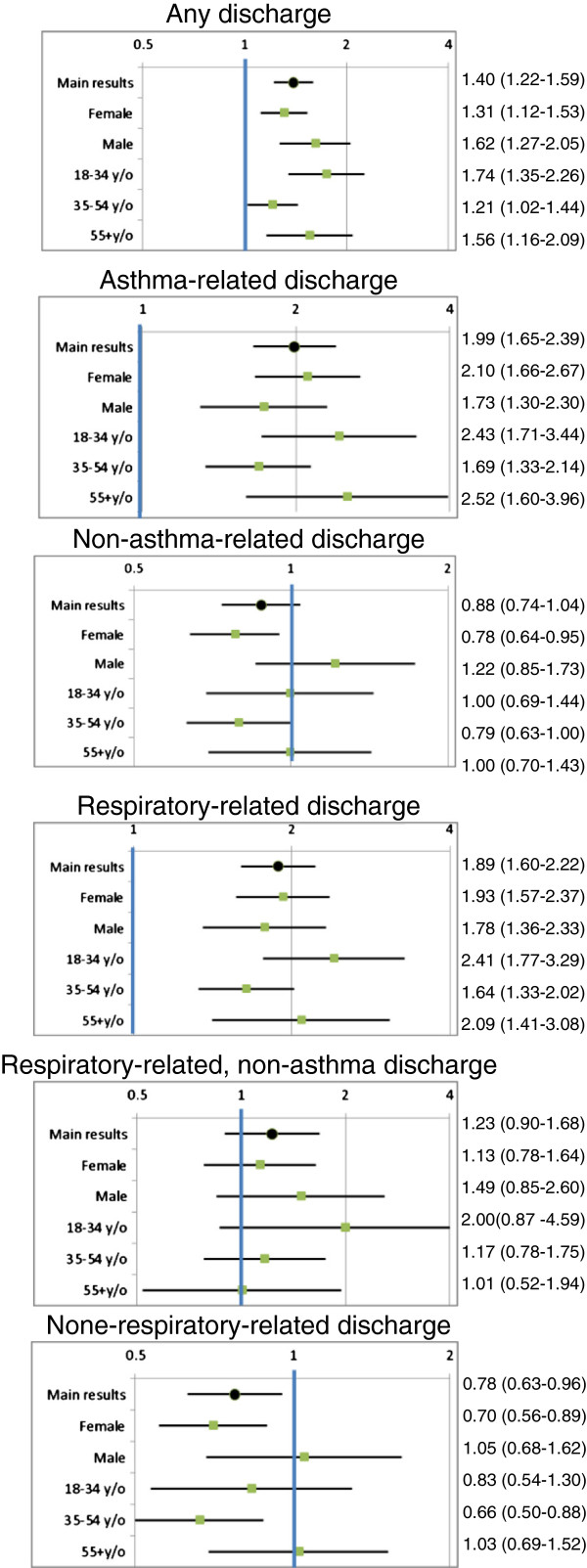
Subgroup analysis*. *All RRs are adjusted for the following variables estimated in the 180 days prior to the start of the time window: number of asthma-related hospital admissions, outpatient services use, medication dispensations, number of dispensations of short-acting beta-agonists, inhaled corticosteroids, and combined inhaled corticosteroids and long-acting beta-agonists, as well as total number of hospital admissions, outpatient services use, medication dispensations, and the Charlson co-morbidity index.

Results of the subgroup analysis are provided in Figure 2. No obvious trend could be observed for the RRs across sex and age groups, although it appears the RRs for asthma-related or respiratory-related outcomes were higher among women than men, whereas the non-asthma-related and non-respiratory-related RRs were reciprocally lower among women compared with men. The negative association between non-respiratory-related discharges and asthma admissions disappeared in several subgroups but persisted among women and individuals 35–54 years old.

### Sensitivity and alternative analyses

**Figure 3:**
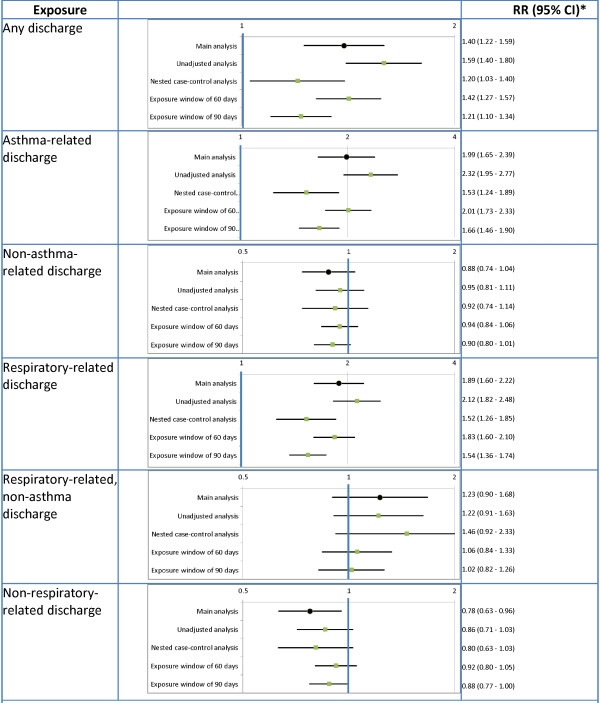
Results of the sensitivity and subgroup analyses. *All RRs, except for the unadjusted case-cross-over, are adjusted for the following variables estimated in the 180 days prior to the start of the time window: number of asthma-related hospital admissions, outpatient services use, medication dispensations, number of dispensations of short-acting beta-agonists, inhaled corticosteroids, and combined inhaled corticosteroids and long-acting beta-agonists, as well as total number of hospital admissions, outpatient services use, medication dispensations, and the Charlson co-morbidity index.

Results of the sensitivity and alternative analyses are provided in Figure 3. For the most part the overall direction and significance of the associations remained the same, with one exception: the negative association between non-respiratory-related discharges and asthma-related admissions disappeared in all sensitivity and alternative analyses.

Overall, in 31.6% of case time windows and 29.1% of control time windows a controller medication was dispensed (P < 0.001 for difference). In exposed time windows (time windows with a hospital discharge), this value was 39.9% (43.9% when the discharge was asthma-related and 35.7% when it was non-asthma-related, all P < 0.001 for the difference compared with unexposed time windows). However, in the sensitivity analysis that was adjusted for the use of controller medication, the RR of the exposure only slightly changed compared with the main analysis [RR = 1.38 (95% CI 1.21 - 1.57)].

## Discussion

We used population-based administrative health data of an entire geographic region to investigate whether discharge from hospital alters the short-term risk of admission due to asthma. As expected, we found that asthma-related and respiratory-related discharges are significant predictors of asthma-related readmission, but non-asthma-related discharges were not associated with the risk of asthma-related readmissions. Other findings of the study remained the same in several sensitivity analyses. Our overall conclusion is that non-respiratory-related admissions do not appear to alter the short-term risk of severe asthma exacerbation requiring admission, whereas an episode of asthma-related discharge is associated with an elevated rate of readmission. In addition to being a chance finding, a reduced risk of asthma-related admission after an episode of non-asthma-related discharge might indicate that not only the underlying risk of asthma exacerbation is not affected by an episode of inpatient care, but such a discharge might result in a higher threshold for readmitting the patient. In addition, a period of inpatient care independent of asthma might prompt a review of asthma management, thus reducing the risk of asthma exacerbation in the post-discharge period. Evidence for this pattern was observed in our data, as dispensation of controller medications were more likely to occur in time windows with, compared with those without, a hospital discharge record.

To our knowledge, the association between discharge from hospital and the short-term risk of admission due to asthma has not previously been investigated. Other investigators have assessed the rate of readmission after discharge from a previous asthma-related hospitalization, and factors altering such rates [6,18-21]. But these studies have not attempted to contrast the risk in the post-discharge period with control periods, and thus have not been able to show any change in the risk. In addition, many of such studies have been based on longer-term follow-up periods, and the associations reflect the impact of post-discharge care rather than the short-term effect of previous hospitalization [6,18].

In addition to relying on the data of an entire geographic region that is free from selection bias, the choice of the case-crossover design gives weight to the validity of our findings. Many factors, not necessarily captured in measures of asthma severity and co-morbidity, can alter the risk of hospitalization for an individual (e.g., threshold for hospitalization in the individual’s local health setting, availability of hospital beds, to name a few) thus causing a spurious association between the exposure and outcome. As such, we believe the case-crossover design, in which such factor are relatively constant within a patient, is a robust design and the main results are less affected by such biases than the results of the nested case control study (although the overall results were similar).

The limitation of our study should be acknowledged. The identification of asthma was based on resource use records. However, the case definition of asthma, used by several other investigators, in combination with a record of asthma-related hospitalization, must have created a very specific sample of asthma patients. Nonetheless, we acknowledge that the diagnostic accuracy of asthma-related hospitalization in the extreme of age groups might not be optimal. Further, the risk of asthma-related readmissions might well be affected by the cause of the previous hospitalization. We decided not to subdivide the exposure any further than asthma-related and respiratory-related conditions as we were concerned multiple-comparison issues would make the interpretation of the results difficult. Additionally, the risk of asthma-related admission might be a function of events occurring within the prior hospitalization, such as whether appropriate care for asthma was provided at discharge, the discharge medications for asthma, and so on. The observed pattern of medication dispensation indicated that around the time of discharge, whether asthma-related or not, patients were more likely to fill prescriptions for asthma controller medications, but in the sensitivity analysis that adjusted for this pattern, no major changes in the findings were observed. This suggests that dispensation of controller medications did not play a major role in the observed findings. Unfortunately, as in many administrative health databases, medication records during inpatient time were not captured in our data, and in the 30-day time window individuals are most likely taking the medications they received during inpatient time and upon discharge; as such, dispensation records could not have been reliably interrogated for such associations.

While we confirmed the previous findings of an elevated risk of readmission after an asthma-related hospitalization, our study indicates that the risk of an asthma-related hospitalization is not increased after discharge from non-asthma-related admission. This is in line with the general belief that the significance of post-hospital syndrome is primarily related to certain co-morbidities of chronic diseases in the older population, and is not a general period of increased risk affecting an inherently inflammatory condition such as asthma. These findings can be of relevance from a policy and clinical perspectives with regard to the managements and recommendations patients with asthma receive upon discharge from hospital. Lack of association between non-asthma-related admissions with a subsequent risk of an asthma-related hospitalizations means in patients with known asthma who are hospitalized due to non-asthma reasons, care providers need to focus on other health conditions that are known to cause readmission. Future research is required to associate the risk of readmission with such factors as the level of asthma care during admission, outpatient care immediately after discharge, and provision of asthma controller medications in the post-discharge period as potentially relevant and modifiable factors determining the risk of asthma-related readmission.

## Competing interests

The authors declare that they have no competing interests.

## Authors’ contribution

MS proposed the overall research question. MS, JMF, and LL conceived the design of the study and participated in planning the detailed analysis plan. LL helped with the acquisition of the data. MS performed the analyses and wrote the first version. All authors read and approved the final manuscript.

## Supplementary Material

Additional file 1: Table S1 List of asthma-related medications. Click here for file
