# Supplementary material for: Post-hospital syndrome in adults with asthma: a case-crossover study
Source: Allergy Asthma Clin Immunol. 2013 Dec 23;9(1):49. doi: 10.1186/1710-1492-9-49 (PMC3880050; doi:10.1186/1710-1492-9-49)
Supplement: Additional file 1: Table S1 — List of asthma-related medications. [file 1710-1492-9-49-S1.pdf]

Additional file 1

All medications are used in calculating the number of asthma-related dispensations as a covariate for asthma severity; but only a subset of the list is used for the case definition (refer to the right-most column)

Medication categories	Active ingredient(s)	Anatomical Therapeutic Chemical (ATC)	Drug Identification Numbers (DIN)	Used in case definition?
**Used in the 'narrow definition' of resource use (short list), also used for case-definition of asthma (see text)**				
Inhaled corticosteroids (ICS)	Beclomethasone	R03BA01	2242030, 2242029, 374407, 828521, 828548, 872334, 893633, 897353, 1949993, 1950002, 2079976, 2213710, 2213729, 2215039, 2215047, 2215055, 2216531	Y
	Budesonide	R03BA02	2229099, 1978918, 1978926, 852074, 851752, 851760	Y
	Fluticasone	R03BA05	2237247, 2237246, 2237245, 2237244, 2244293, 2244292, 2244291, 2174731, 2174758, 2174766, 2174774, 2213583, 2213591, 2213605, 2213613	Y
	Ciclesonide	R03BA08	2285614, 2285606, 2303671	Y
Short-acting beta-agonists (SABA)	Salbutamol	R03AC02	790419, 812463, 832758, 832766, 851841, 860808, 867179, 897345, 1926934, 1938851, 1938878, 1945203, 1947222, 1986864, 2022125, 2046741, 2048760, 2069571, 2084333, 2148617, 2154412, 2173360, 2208229, 2208237, 2208245 2212315, 2212323, 2213400, 2213419, 2213427, 2213478, 2213486, 2214997, 2215004, 2215616, 2215624, 2215632, 2216949, 2231430, 2231488, 2231678, 2231783, 2231784, 2232570, 2232987, 2236931, 2236932, 2236933, 2239365, 2239366, 2241497, 2243115, 2243828, 2244914, 2245669, 2259583, 2326450	Y
		R03CC02	620955, 620963, 874086, 894249, 894257, 1932691, 2035421, 2063689, 2091186, 2146843, 2146851, 2164434, 2164442, 2165368, 2165376, 2212390, 2213435, 2213443, 2213451, 2261324	Y
	Terbutaline	R03AC03	786616	Y
	Orciprenaline	R03CB03	249920, 3891, 2236783, 2229862, 2152568, 2192675	Y
Long-acting beta-agonists (LABA)	Salmeterol	R03AC12	2211742, 2214261, 2231129, 2136139, 2136147	Y
	Formoterol	R03AC13	2230898, 2237224, 2237225	Y
ICS and LABA in combination (ICS+LABA)	Budesonide, formoterol	R03AK07	2245385, 2245386	Y

	Fluticasone, salmeterol	R03AK06	2240835, 2245126, 2245127, 2240836, 2240837	Y
Leukotriene receptor antagonists (LTRA)	Montelukast	R03DC03	2247997, 2238217, 2243602, 2238216	Y
	Zafirlukast	R03DC01	2236606	Y
	~~Zileuton~~	~~Not available in Canada~~		
Anti-immunoglobulin E monoclonal antibody	Omalizumab	R03DX05	2260565	Y
Inhaled mast cell stabilizers	Cromoglicic acid (cromolyn)	R03BC01	2231431, 2231671, 2046113, 534609, 555649, 261238, 638641, 2049082, 2219468	Y
Theophylline	Choline theophyllinate	R03DA02	346071, 405310, 441724, 441732, 451282, 458708, 458716, 476366, 476390, 476412, 503436, 511692, 536709, 565377, 589942, 589950, 792934	Y
	Theophylline	R03DA04	156701, 261203, 460982, 460990, 461008, 466409, 488070, 532223, 556742, 575151, 599905, 627410, 631698, 631701, 692689, 692697, 692700, 722065, 1926586, 1926594, 1926608, 1926616, 1926640, 1966219, 1966227, 1966235, 1966243, 1966251, 1966278, 1966286, 2014165, 2014181, 2230085, 2230086, 2230087	Y
	Aminophylline	R03DA05	14923, 178497, 497193, 497193, 497207, 582654, 582662, 868450, 2014270, 2014289	Y
Inhaled anticholinergics	Ipratropium bromide	R01AX03	2246084, 2246083, 2163705, 2163713, 2240508, 2240072	N
		R03BB01	2126222, 2243827, 2231494, 731439, 576158, 2247686, 824216, 2026759, 1950681, 2239131, 2216221, 2210479, 2231785, 2236934, 2236935, 2237134, 2237135, 2239627, 2231135, 2231136, 2231245, 2231244, 2097141, 2097176, 2097168	N
	Ipratropium bromide, fenoterol	R03AK03	02148633	N
	Tiotropium bromide	R03BB04	02246793	N
Other beta-agonists	Epinephrine	R03AA01	2017555, 466417, 525103, 1927582	N
	Ephedrine	R03CA02	2237085, 2229698, 2100231, 2100258, 2243148, 2236722, 2229678, 2219743, 2012111, 2229711, 38121, 2242961, 876534, 893323, 893331, 438847, 2242639, 2126419, 2126400	N
	Isoprenaline	R03AB02	2017652	N
	Orciprenaline	R03AB03	1923870, 1928449, 2017660, 254134, 3859	N
Other corticosteroids	Cortisone	H02AB10	280437, 16241, 16446, 16438	N
	Triamcinolone	H02AB08	2194090, 15016, 15024, 2194082	N

	Prednisone	H02AB07	610623, 598194, 550957, 312770, 252417, 210188, 868426, 868434, 868442, 21695, 232378, 607517, 508586, 156876, 271373, 271381	N
	Prednisolone	H02AB06	21679, 2230619, 2152541, 2245532	N
	Methylprednisolone	H02AB04	1934325, 1934333, 1934341, 30759, 30767, 36129, 30988, 2245406, 2245400, 2245408, 2245407, 2241229, 2231893, 2231894, 2231895, 2232750, 2232748, 2063727, 2063697, 2063719, 2063700, 36137, 2230210, 2230211, 30678, 30651, 30643	N
	Betamethasone	H02AB01	2237835, 36366, 2063190, 176834, 28096, 28185	N
	Hydrocortisone	H02AB09	888222, 888230, 888206, 888214, 30910, 30929, 872520, 872539, 878618, 878626, 30635, 30600, 30619, 30627	N
	Dexamathasone	H02AB02	2261081, 2250055, 213624, 16462, 354309, 716715, 874582, 1977547, 664227, 2204274, 2204266, 295094, 285471, 489158, 2239534, 732893, 732885, 2260301, 2237044, 2260298, 2237046, 2237045, 1946897, 1964976, 1964968, 1964070, 2279363, 783900, 751863, 2311267, 2240687, 2240685, 2240684	N
Other xanthines	Theophylline, combination	R03DA54	545090, 476374, 334510, 356123, 792942, 721301, 317225, 828718, 640093, 828726, 828742, 307548	N
Other anti-allergic agents	Levocabastine	R01AC02	2020017	N
	Ketotifen	R06AX17	2221330, 2176084, 2230730, 2218305, 2231680, 2231679, 600784, 577308	N
